# Luciferase-Based Screen for Post-translational Control Factors in the Regulation of the Pseudo-Response Regulator PRR7

**DOI:** 10.3389/fpls.2019.00667

**Published:** 2019-05-22

**Authors:** Yeon Jeong Kim, David E. Somers

**Affiliations:** Department of Molecular Genetics, The Ohio State University, Columbus, OH, United States

**Keywords:** circadian clock, pseudo-response regulator, PRR7, ELF3, ELF4, EMS mutagenesis, post-translational regulation

## Abstract

Control of protein turnover is a key post-translational control point in the oscillatory network of the circadian clock. Some elements, such as TOC1 and PRR5 are engaged by a well-described F-box protein, ZEITLUPE, dedicated to their proteolytic turnover to shape their expression profile to a specific time of night. For most other clock components the regulation of their protein abundance is unknown, though turnover is often rapid and often lags the cycling of the respective mRNA. Here we report the design and results of an unbiased genetic screen in Arabidopsis to uncover proteolytic regulatory factors of PSEUDO-RESPONSE REGULATOR 7 (PRR7), a transcriptional repressor that peaks in the late afternoon. We performed EMS mutagenesis on a transgenic line expressing a *PRR7::PRR7-luciferase* (*PRR7-LUC*) translational fusion that accurately recapitulates the diurnal and circadian oscillations of the endogenous PRR7 protein. Using continuous luciferase imaging under constant light, we recovered mutants that alter the PRR7-LUC waveform and some that change period. We have identified novel alleles of *ELF3* and *ELF4*, core components of the ELF3-ELF4-LUX Evening Complex (EC), that dampen the oscillation of PRR7-LUC. We report the characterization of two new hypomorphic alleles of *ELF3* that help to understand the relationship between molecular potency and phenotype.

## Introduction

The circadian clock system helps to coordinate daily oscillations in gene expression, metabolism and physiology to help optimize growth and reproduction under daily light/dark cycles. It is primarily comprised of interlocked autoregulatory feedback loops of gene transcription and translation, but relies strongly on numerous post-transcriptional and post-translational processes (Seo and Mas, 2014; Mateos et al., 2018). In Arabidopsis, one of the core loops involves the evening-expressed gene TIMING OF CAB EXPRESION 1 (TOC1) and the morning expressed genes CIRCADIAN CLOCK ASSOCIATED 1/LATE ELONGATED HYPOCOTYL (CCA1/LHY) which act together in a mutually repressive negative feedback.

Among the additional transcriptional repressors/co-repressors and activators/co-activators that comprise a fully functional clock is a five-member family of pseudo-response regulators (PRRs). PRR9, PRR7, PRR5, PRR3, and TOC1 are expressed in sequential and overlapping order over the course of diel and circadian cycles (Matsushika et al., 2000; Fujiwara et al., 2008). Numerous studies have highlighted the dual role that most of these PRRs play as transcriptional repressors (Farre and Liu, 2013). At one level they act to repress transcription of certain core clock genes, helping to maintain the correct period and robustness of the central oscillator. In particular, the waveform of CCA1/LHY expression is established by the sequential and ordered expression, from morning to evening, of PRR9, PRR7, and PRR5, which results in the direct repression of these morning genes at all times except for early morning and late night (Nakamichi et al., 2010). At the same time, the precise phase-specific expression of each of the PRRs contributes to an orchestration of concomitant specific phasing of output gene expression (Nakamichi et al., 2012; Farre and Liu, 2013; Liu et al., 2013, 2016).

PSEUDO-RESPONSE REGULATOR7 (PRR7) is a key component in the control of the plant circadian clock. It is one of five closely related transcriptional repressors in the Arabidopsis clock that controls not only the period of the oscillator, but also acts on core genes involved in abiotic stress (Liu et al., 2013; Kolmos et al., 2014). PRR7 occupies a unique, synergistic position in the plant circadian system: the *prr7* mutant (ca. + 1 h) enhances the short period of the *prr5* mutant (−1.5 h) to a much shorter period (*prr5 prr7* = −5.0 h), while it also strongly enhances the long period of the *prr9* mutant (+1.5 h) to be even longer (*prr9 prr7* = +8 h) (Farre et al., 2005; Mizuno and Nakamichi, 2005; Nakamichi et al., 2005; Salome and McClung, 2005). These findings show that PRR7 operates centrally and together with other PRR proteins to control period, but how this occurs is unknown. PRR7 and other PRRs also act with the co-repressor TOPLESS (TPL) and histone deacetylases to form repressive complexes (Wang et al., 2013).

PSEUDO-RESPONSE REGULATOR7 also plays a central role in the abiotic stress response. PRR7 is involved in the regulation of ABA-related processes, including control of genes affecting salt and freezing tolerance. A high percentage (28%) of PRR7 targets are also ABA-regulated, with more than one third of PRR7 target genes possessing ABA-responsive elements (Liu et al., 2013). STO (AT1G06040; SALT TOLERANCE), STH (AT2G31380 salt tolerance homolog), and members of the CBF/DREB family (AT4G25470, AT4G25490, AT4G25480) are examples of genes targeted by PRR7 that are involved in salt, drought, and cold stress tolerance (Nakamichi et al., 2012; Liu et al., 2013).

Given this central role for PRR7, and since the post-translational regulation of only two PRR family members has been well characterized (TOC1 and PRR5), we undertook a forward genetic screen to identify PRR7 protein turnover factors. A previous luciferase-based screen successfully identified ZEITLUPE (ZTL) as an F-box protein responsible for the E3 ligase-based proteolysis of TOC1 and PRR5 (Mas et al., 2003; Kiba et al., 2007; Fujiwara et al., 2008). The rapid protein turnover of the clock-related PRRs (Fujiwara et al., 2008; Nakamichi et al., 2010) suggests dedicated proteolytic factors may be associated with each to ensure their proper phasing during the circadian cycle.

Our approach employed a PRR7-luciferase translational fusion (*PRR7::PRR7-LUC*) and EMS mutagenesis to identify plants with aberrantly high levels of luminescence at times when PRR7 levels are normally low. We recovered multiple classes of factors that alter the luminescence profile, and characterized here are three new alleles of EARLY FLOWERING 3 (*ELF3*) identified from the screen.

## Materials and Methods

### Plasmid Construction and Transgenic Plant

To generate the *PRR7::PRR7-luciferase* (*PRR7::PRR7-LUC*) transgenic line, *PRR7* coding sequence from ATG to STOP codon was subcloned into *Nco I* site in pPZP-BAR DONR plasmid harboring luciferase fused to 1208 bp of the PRR7 promoter, which was kindly provided by the McClung laboratory (Dartmouth College, Hanover, NH, United States). A genomic fragment containing 2223 bp upstream of the 5′ end of *PRR7* was then cloned into *EcoRV* site upstream of the *PRR7* gene to replace the 1208 bp-promoter resulting in *PRR7::PRR7-LUC* (Nakamichi et al., 2010). *Arabidopsis thaliana* plants (Col-0) were transformed with *Agrobacterium tumefaciens* strain GV3101 by a floral dip method (Clough and Bent, 1998). Basta-resistant primary transformants were self-pollinated, and a high amplitude cycling bioluminescence homozygous line was selected from the T_3_ generation. After validating that the circadian oscillation in luciferase activity correlated with the abundance of PRR7-LUC protein, T_4_ seeds were harvested and used for ethyl methanesulfonate (EMS) mutagenesis. To construct TAP-tagged ELF3^WT^, ELF3^A37T^, and ELF3^P666S^, the DNA fragment containing the nucleotide substitution corresponding to the mutation was subcloned to pENTR/D-TOPO (Invitrogen, K240020) and verified by sequencing. The TAP tag (2x Protein A IgG binding domain His-9x myc) was placed at the N-terminus of ELF3 by LR recombination with pN-TAPa (Rubio et al., 2005). HA-tagged-ELF4, LUX, GI, and PIF4 were obtained by cloning pENTR/D-TOPO clones into pCsVMV-HA-C-1300 vector. pENTR4-phyB was kindly supplied by the Quail laboratory (UC Berkeley, CA) and cloned into pCsVMV-GFP-N-1300 vector. GFP-TOC1 construction was described previously (Wang et al., 2013). Primers for plasmid construction are listed in Supplementary Table S1.

### EMS Mutagenesis

Approximately 27,500 *PRR7::PRR7-LUC* seeds were EMS treated. Briefly, the seeds were soaked overnight in 0.1 % potassium chloride and transferred to 100 mM phosphate buffer containing 0.25 % EMS. After shaking incubation at room temperature for 15 h, the seeds were rinsed three times with 100 mM sodium thiosulfate and washed several times in water. The mutagenized seeds were sown on 10 soil flats and stratified at 4°C for 4 days, and grown until seed set under 16L:8D at 22°C. Flats were harvested as 256 pools of between 50–150 plants/pool.

### Bioluminescence Assays

Approximately one thousand and two hundred seeds from each pool were plated on Murashige and Skoog (MS) media containing 3 % sucrose and grown in 12:12 LD white-light cycles (50 μmol m^−2^s^−1^) for 5 days. Seedlings were sprayed with 1 mM luciferin solution containing 0.01 % Triton X-100 and transferred to imaging chamber. Images were obtained with an Andor iKon-M 934 CCD camera (Andor Technology, Belfast, United Kingdom) for 5 min every 2 h under continuous LED red and blue light (30 μmol m^−2^s^−1^) at 22°C. Luminescence signals were quantified by Image-J software.

### Phenotypic Analyses

For flowering time measurement, dried seeds were sterilized and sown on soil flats followed by stratification at 4°C in dark for 4 days. Plants were grown at 22°C under a 16L:8D photoperiod (white light, 110 μmol m^−2^s^−1^) and watered as necessary until the plants were flowered. The number of rosette leaves were determined from the plants when the bolt reached 1 cm. For hypocotyl length analysis, surface sterilized seed were plated on MS media without sucrose and stratified at 4°C in dark for 4 days. Germination and growth were carried out at 22°C in continuous red LED light with different light intensities ranging from 0.52 to 20.32 μmol m^−2^s^−1^. Hypocotyl length was measured from images of the seedlings 4 days after illumination using Image-J software.

### RNA Extraction and Quantification Real-Time PCR

Total RNA was extracted using Trizol^TM^ reagent according to manufacturer’s protocol (Thermo Fisher Scientific, Cat #155960-028) and treated with RNase-free DNase I (Ambion, Cat #AM2224) for 30 min at 37°C. First-strand cDNA was synthesized from 3 μg of the total RNA using Oligo(dT)_12-18_ primer and SuperScript^TM^ III reverse transcriptase (Thermo Fisher Scientific, Cat #18080093) followed by RNase H treatment for 20 min at 37°C. For quantitative real-time RT-PCR, specific primers and equal volume of the template cDNA were combined with 7.5 μL iQ^TM^ SYBR-Green Super Mix (Bio-Rad, Cat #1708882), and subjected to following thermal cycling conditions: 94°C for 2 min; followed by 44 cycles of 94°C for 15 s and 55°C for 34 s. The quantities of input cDNA were normalized to *At5g15400*, and transcript levels of target genes were analyzed by CFX Manager^TM^ Software (Bio-Rad). Primers used for qRT-PCR are listed in Supplementary Table S1.

### Protein Extraction and Immunoblotting

Protein extraction and immunoblotting were performed as described previously (Kim et al., 2003). Briefly, ground tissues were homogenized with extraction buffer (50 mM Tris–Cl, pH 7.5, 150 mM NaCl, 0.5% Nonidet P-40, 1 mM EDTA, 3 mM dithiothreitol, 1 mM phenylmethylsulfonyl fluoride, 5 μg/ml leupeptin, 1 μg/ml aprotinin, 1 μg/ml pepstatin, 5 μg/ml antipain, 5 μg/ml chymostatin, 50 μM MG132, 50 μM MG115, and 50 μM ALLN) and centrifuged at 16,000 *g* at 4°C for 10 min. To detect luciferase protein, supernatant proteins were concentrated by TCA precipitation and resultant pellets were resuspended in Urea/SDS loading buffer (40 mM Tris–Cl, pH 6.8, 8 M Urea, 5% SDS, 1 mM EDTA, 2% 2-mercaptoethanol). The total proteins were separated on a 8% SDS-PAGE gel (acrylamide:bisacrylamide, 37.5:1) and probed with 1:1,000 anti-luciferase (Sigma, L0159) and 1:15,000 anti-ADK antibody (gift from Dr. David Bisaro), as a loading control. For immunoprecipitated proteins, 1:2,000 anti-myc (Sigma, M4439), 1:1,000 anti-HA (Sigma, 3F10), and 1:5,000 anti-GFP (Abcam, ab6556) were used.

### Coimmunoprecipitation

*Agrobacterium tumefaciens* strains *GV3101* or *AGL-1* harboring TAP-ELF3^WT^, TAP-ELF3^A37T^, or TAP-ELF3^P666S^, and HA-ELF3^WT^, HA-ELF3^A37T^, HA-ELF3^P666S^, HA-ELF4, HA-LUX, HA-GI, GFP-TOC1, HA-PIF4, or phyB-GFP were co-infiltrated into *Nicotiana benthamiana* leaves. Total proteins were extracted from the leaves collected 2–3 days after the infiltration, and the cleared supernatant was incubated with 20 ul of Human IgG-Agarose (Sigma, A6284) for 1 h at 4°C. After washing the resin with cold extraction buffer 4–5 times, 1.5 ul of HRV3C protease (Thermo Scientific, 88947) was added for1.5 h at 4°C to release the resin-bound immune complexes, and separated by SDS-PAGE.

### Statistical Analysis

For comparison between the plant groups, one-way Analysis of variance (ANOVA) followed by Tukey’s HSD test was applied using R 3.5.0. Statistically significant differences (*p* < 0.05) are represented by small letters within the figures.

## Results

### Identification of Factors Altering *PRR7::PRR7-LUC* Expression

**FIGURE 1:**
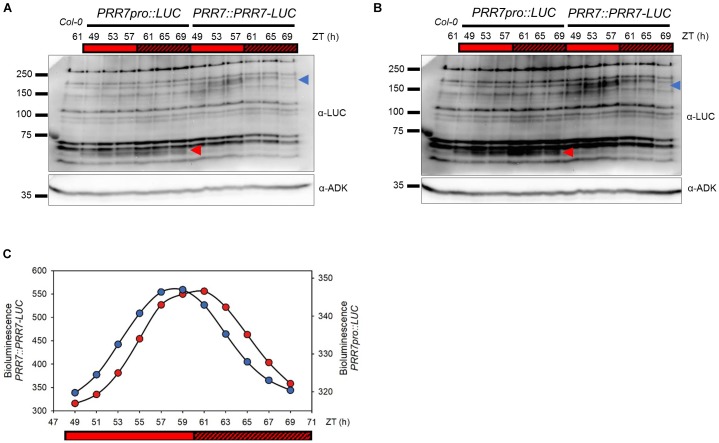
Cycling pattern of PRR7-LUC protein abundance in *PRR7::PRR7-LUC*. **(A)** Image of immunoblotting of luciferase and PRR7-LUC in *PRR7pro::LUC* and *PRR7::PRR7-LUC*, respectively. 11-days old seedlings grown in 12L:12D white-light cycles were released to continuous red light (50 μmol m^−2^s^−1^) and collected at indicated ZT time points. Protein abundances of luciferase (red arrowhead) and PRR7-LUC (blue arrowhead) were analyzed by anti-LUC immunoblotting after TCA protein precipitation. ADK protein was used as a loading control. **(B)** Longer exposure image of immunoblotting. **(C)** Relative luminescence intensity of luciferase (*PRR7pro::LUC, red*) and PRR7-LUC (*PRR7::PRR7-LUC, blue*). 7-day old seedlings grown under same conditions as used in immunoblotting were subjected to luminescence imaging analysis. PRR7-LUC is indicated by the blue arrow **(A)** showing a cycling pattern consistent with luminescence signals.

**FIGURE 2:**
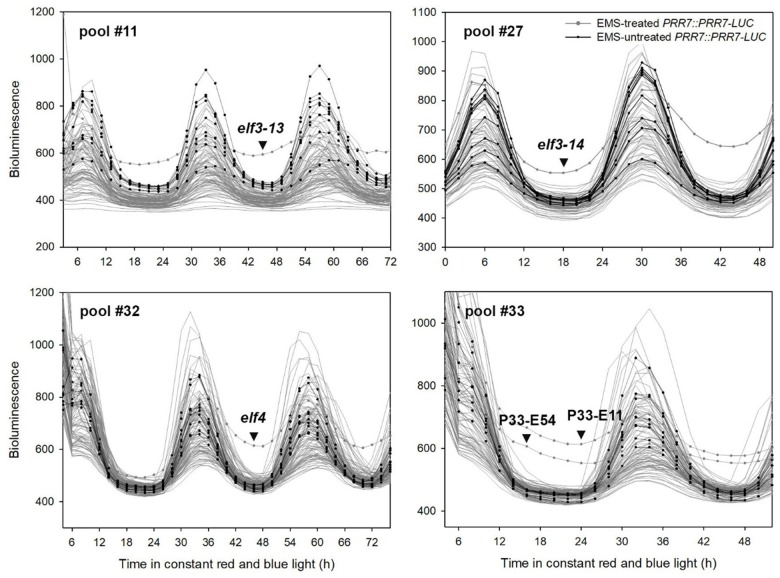
Representative PRR7 turnover factor candidates. The EMS-mutagenized *PRR7::PRR7-LUC* M_2_ seedlings were grown on MS media in 12L:12D white-light cycles for 5 days and transferred to continuous red and blue light (30 μmol m^−2^s^−1^) for imaging. Luminescence signals obtained from seedlings grown on the same MS plate were plotted together. Non-EMS-treated *PRR7::PRR7-LUC* seedlings were included to estimate variability of luminescence signals of the M_2_ seedlings. The selected candidates showing increased PRR7-LUC signal are indicated by an arrowhead. Candidates from pool #33 remain unidentified.

We identified a *PRR7::PRR7-luciferase* (*PRR7-LUC*) translational fusion line to perform an EMS-based mutant screen for PRR7 turnover factors. We chose a transgenic line in which the circadian oscillation of the PRR7-LUC protein recapitulates endogenous PRR7 phasing (Figure 1A,B), and which also demonstrates robust circadian oscillations in luminescence (Figure 1C). We reasoned that mutants exhibiting luminescence oscillations with reduced amplitude and/or higher trough levels would be candidates for a loss-of-function in factors involved in PRR7-LUC turnover. 41,433 EMS-mutagenized seedlings from 44 pools (50–150 plants/pool) were screened and 31 candidates were identified. Some mutants show essentially WT period but with significantly higher troughs (Figure 2), as expected if PRR7 proteolysis is reduced. Before further mapping, we first tested whether any mutant loci corresponded to known clock loci. In particular, loss-of-function mutations in evening complex (EC) genes (ELF3, ELF4, and LUX) cause circadian arrhythmia and upregulation of PRR7 transcription (Kolmos et al., 2009; Dixon et al., 2011; Kolmos et al., 2011; Mizuno et al., 2014; Choudhary et al., 2015; Huang and Nusinow, 2016).

We extracted genomic DNA from the 20 surviving lines with increased *PRR7-LUC* luminescence and examined the genomic sequences of EC genes. Fifteen of twenty lines, originating from seven different pools, had single point mutations in *ELF3* or *ELF4* coding regions, causing amino acid substitutions or predicted premature translation termination. The remaining five lines have no mutations in the EC genes, and segregate as single gene mutations in backcrossed F2 populations. Two of these lines showed growth and development similarities to the *GIGANTEA* (*GI*) mutant, *gi-2* (long period in constant light and late flowering). We sequenced *GI* and confirmed that the mutations are not at that locus.

**FIGURE 3:**
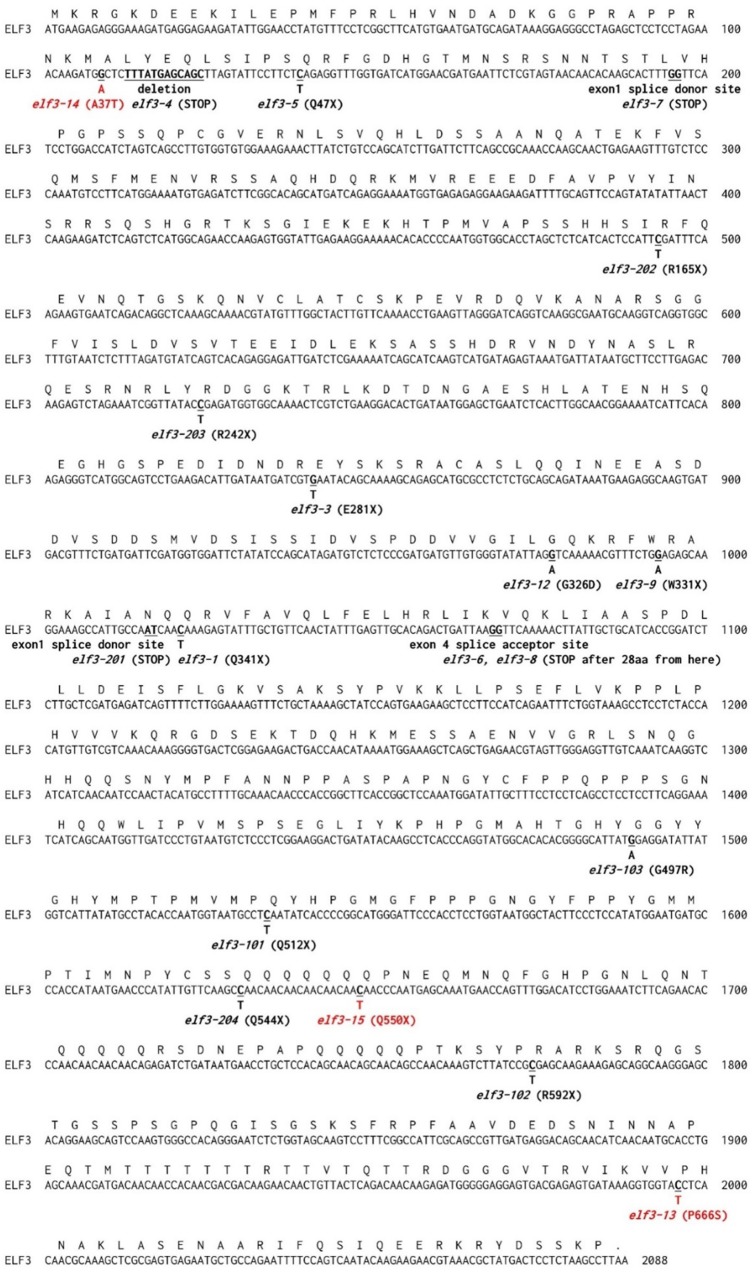
Protein coding sequence of *ELF3*. Deduced amino acid sequences and nucleotide changes in known *elf3* alleles are shown above and below the nucleotide sequence. Sites of novel mutants identified in this study are indicated in red. Sources for the known indicated *elf3* alleles are: *elf3-1*, *elf3-3*, *elf3-4*, *elf3-5*, *elf3-6*, *elf3-7*, *elf3-8*, and *elf3-9* (Hicks et al., 2001); *elf3-12* (Kolmos et al., 2011); *elf3-101*, *elf3-102*, and *elf3-103* (Yoshida et al., 2009); and *elf3-201*, *elf3-202*, *elf3-203*, and *elf3-204* (Kinoshita et al., 2011).

Ten of the fifteen EC mutants showed single amino acid substitutions in *ELF3* as either proline to serine (P666S; *elf3-13*), alanine to threonine (A37T; *elf3-14*), or premature termination (Q550X; *elf3-15*) (Figure 2, 3). These mutations occurred at the N-terminal and C-terminal regions of ELF3, respectively, which are highly conserved among plant ELF3 orthologs (Supplementary Figure S1). The *elf4* mutant (Figure 2, pool #32) is a presumed null (Trp26 STOP; TGG – >TAG).

### Effects of Novel *elf3* Mutations on Growth and Development

Each of these mutations co-segregated with high *PRR7-LUC* luminescence in backcrossed F2 populations. Higher levels of PRR7-LUC were also detected in the mutants at ZT1, confirming that luminescence levels arose from more PRR7-LUC protein accumulation (Supplementary Figure S2). To identify the effects of these novel mutations on growth and development, we backcrossed four independently isolated *elf3* mutant lines (*elf3-13#1* and *elf3-13#2*; *elf3-14#1*, and *elf3-14#2*) to *PRR7::PRR7-LUC*/Col-0 and selected one individual segregant (BC1F3) from the respective F2 populations to characterize. We included Q550X premature translation termination mutants, (*elf3-15#1* and *elf3-15#2* BC1F3 segregants) as a controls along with the null, *elf3-8* (Hicks et al., 2001).

**FIGURE 4:**
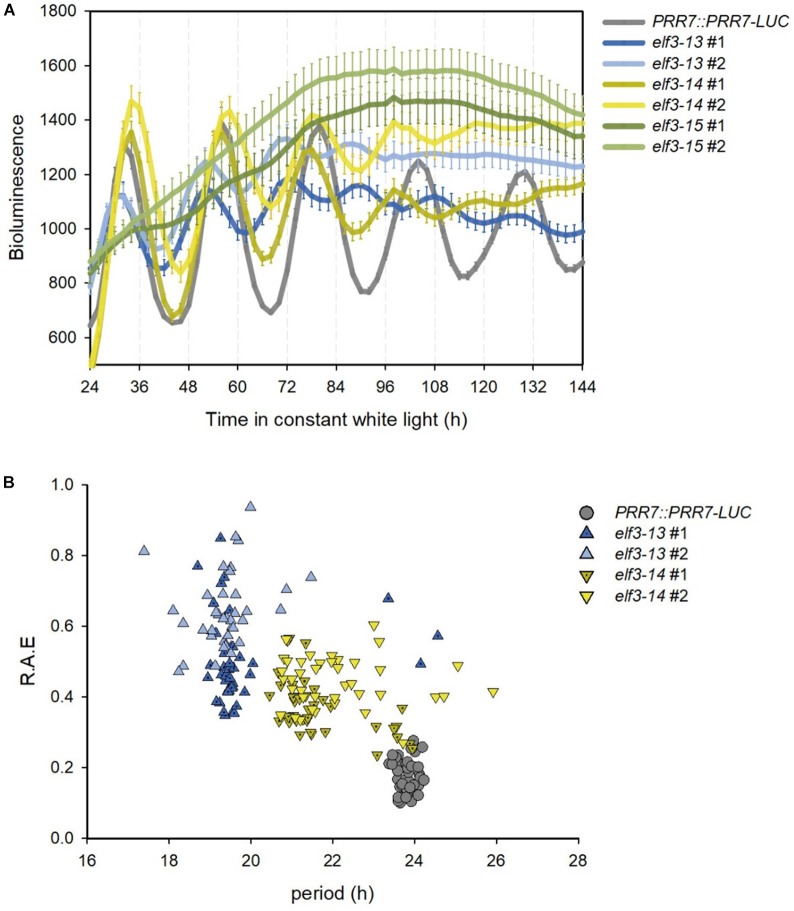
Effects of novel *elf3* mutations on circadian period. 7-day old seedlings of *PRR7::PRR7-LUC*, *elf3-13*, *elf3-14*, and *elf3-15* were entrained in 12L:12D white-light cycles, then moved to continuous white light (50 μmol m^−2^s^−1^) and luminescence images obtained from ZT2 every 2 h for 7 days. **(A)** Free-running bioluminescence profile of *PRR7::PRR7-LUC* activity. Mean of luminescence values shown with error bars corresponding to SEM (*n* > 37). **(B)** Period versus relative amplitude error (R.A.E).

Severe mutants of *ELF3* result in arrhythmicity or near arrhythmicity in circadian oscillations of gene expression and bioluminescent reporters (Hicks et al., 1996; McWatters et al., 2000; Covington et al., 2001; Kim et al., 2005), while reduced function alleles shorten period (Kolmos et al., 2011). Both *elf3-13* and *elf3-14* shorten period significantly (*elf3-13*: 19.2–19-6 h; *elf3-14*: 21.6–22.0 h; and WT: 23.8 h) while *elf3-15* is arrhythmic (Figure 4A). Using relative amplitude error (R.A.E.) as a measure of oscillation robustness (low values indicate strong rhythms, >0.6 poorly rhythmic or arrhythmic) *elf3-13*, with the shortest period, is more strongly compromised in function than *elf3-14* (Figure 4B).

**FIGURE 5:**
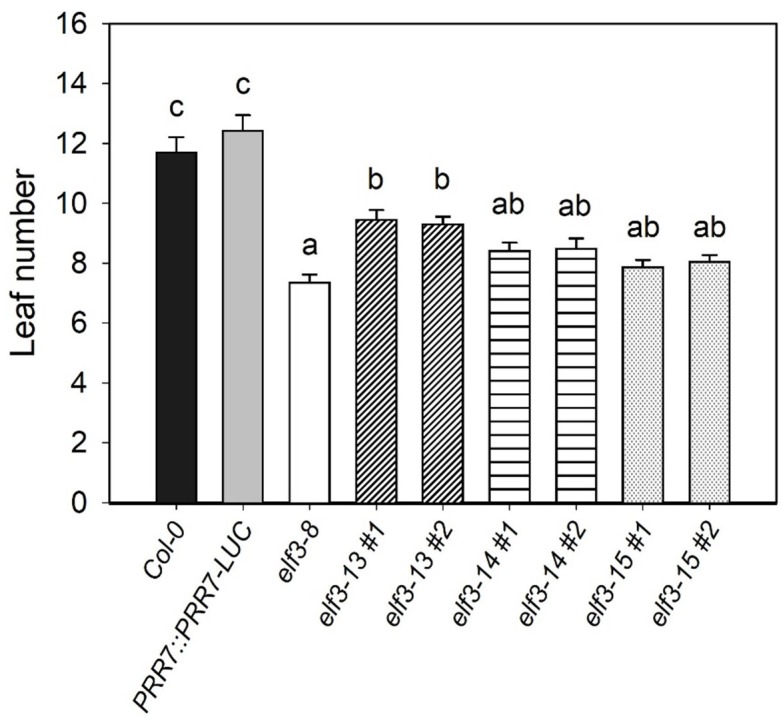
Effect of *elf3* mutations on flowering time. The *Col-0*, *elf3-8*, *elf3-13*, *elf3-14*, and *elf3-15* were grown on soil under long days (16L:8D white-light cycles). Leaf count taken at time of 1 cm bolt. Two individual segregating lines from backcrossed progeny were selected for each *elf3* mutant line. Error bars indicate ± SEM (*n* = 3).

EARLY FLOWERING 3 (*ELF3*) loss-of-function mutations cause early flowering under long and short days (Zagotta et al., 1996; Hicks et al., 2001). The *elf3-15* mutation results in significantly early flowering under long days (16:8) compared to wild-type (*PRR7::PRR7-LUC*/Col-0), but later than *elf3-8* (Figure 5). The very slightly later flowering of *elf3-15*, relative to *elf3-8* may be due to mild overexpression of PRR7 from the presence of the *PRR7::PRR7-LUC* transgene. *elf3-13* also shortened flowering time, but was the least severe allele, compared to *elf3-14*, and the presumed null, *elf3-15*.

**FIGURE 6:**
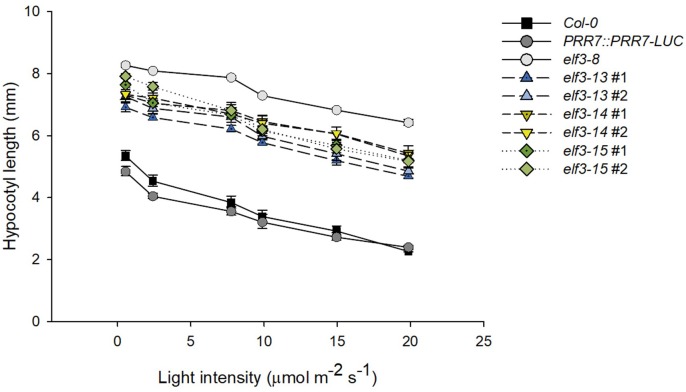
Effect of *elf3* mutations on hypocotyl growth. Col-0 WT, *PRR7::PRR7-LUC* (parent line) *elf3-8*, *elf3-13*, *elf3-14*, and *elf3-15* seeds were plated on MS medium without sucrose stratified (4°C; 4 days) then grown under continuous red light at the indicated light intensities. Error bars indicate ± SEM (*n* = 3).

Light-dependent hypocotyl elongation is a sensitive measure of the extent to which the phototransduction pathway is intact (Gommers and Monte, 2018). Strong *elf3* mutants show diminished light responsiveness, resulting in longer hypocotyls compared to wild-type (Zagotta et al., 1996; Reed et al., 2000). All three *elf3* alleles cause significantly longer hypocotyls under a range of red light intensities (Figure 6). *elf3-15* is the most severe, especially at very low light intensities, with hypocotyl lengths similar to the *elf3-8* null mutant. However, at higher light intensities *elf3-14* is similar to *elf3-15*. *elf3-13* retains the most functionality at all light intensities, relative to the other two alleles, but shows strongly diminished function.

### *elf3-13 and elf3-14* Mutants Retain Some Repressive Functions

**FIGURE 7:**
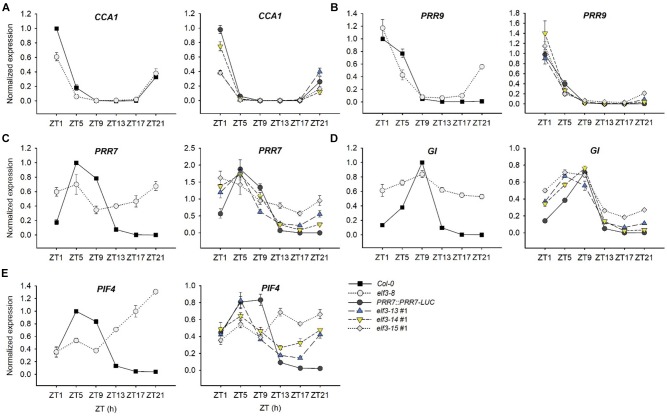
Transcript abundance patterns of select clock genes in *elf3* mutants. 11-day old seedlings were collected from ZT1 to ZT21 every 4 h under 12L:12D white-light cycles. Input RNA was normalized to a *U-box* gene (At5g15400) and expression levels of each transcript were standardized to the peak levels in *Col-0* set to 1. **(A)** Transcript profile of *CCA1*. **(B)** Transcript profile of *PRR9*. **(C)** Transcript profile of *PRR7*. **(D)** Transcript profile of *GI*. **(E)** Transcript profile of *PIF4*. Error bars show SEM from three biological trials.

To further refine our understanding of the effects of these mutations on circadian and stem elongation processes, we examined in the *elf3-13, -14*, and *-15* mutants the expression patterns and levels of select components of both processes known to be under ELF3 control (Figure 7). ELF3 is expressed at night under LD (12 h light:12 h dark) cycles and during subjective night under free-running constant light conditions (Nusinow et al., 2011; Flis et al., 2015). It represses *PRR9* and *PRR7* expression during those times, restricting their expression to early in the day (Dixon et al., 2011; Flis et al., 2015). The *elf3* null (*elf3-8*) shows markedly higher expression levels of both genes during the dark, particularly for *PRR7* (Figure 7B,C). *elf3-15* is similar to *elf3-8*, suggesting it effectively acts as a null, consistent with its circadian arrhythmicity (Figure 4). *elf3-13* and *elf3-14* show a nearly normal expression pattern for PRR9 under LD, but a marked de-repression of PRR7 expression is seen for both mutants during the late night, though not as strongly as for *elf3-15* (Figure 7B,C).

The sequential temporal expression of the PRR proteins contributes strongly to restricting *CCA1* expression to the very late night and early morning (Nakamichi et al., 2010). *ELF3* upregulates *CCA1* indirectly, through repression of the repressors of *CCA1*, *PRR9*, and *PRR7* (Dixon et al., 2011; Kolmos et al., 2011). As a result, *elf3* null mutants (*elf3-8*) show lower peak *CCA1* levels, and *elf3-15* is very similar (Figure 7A; Dixon et al., 2011). *elf3-13* and *elf3-14* appear intermediate in effect, consistent with their effects on *PRR9* and *PRR7* expression (Figure 7A,B).

*GIGANTEA* (*GI*) is a key component in the control of flowering time and numerous other processes (Fowler et al., 1999; Mishra and Panigrahi, 2015), and a co-chaperone in the maturation of the F-bpx protein, ZEITLUPE (ZTL) (Cha et al., 2017). *GI* expression is strongly upregulated in *elf3* null mutants (Fowler et al., 1999; Kolmos et al., 2011; Dixon et al., 2011). We confirm those findings (Figure 7D) and show that *elf3-15* is very similar to *elf3-8* in de-repressing GI at night. Similar to our findings for *PRR7*, both *elf3-13* and *elf3-14* can repress *GI* expression at night, but *elf3-13* is less effective (Figure 7D).

Phytochrome Interacting Factors (PIFs) play multiple, interacting and integrative roles in plant development (Leivar and Monte, 2014). *PIF4* and *PIF5* are clock-regulated at the transcript level, and light-regulated at the protein level, acting as integrators of these signals in the control of hypocotyl elongation (Nozue et al., 2007; Lorrain et al., 2008; Niwa et al., 2009). In the context of the EC complex, ELF3 represses *PIF4* expression at night, which rises strikingly in *elf3* null mutants, including *elf3-15* (Nusinow et al., 2011; Lu et al., 2012; Figure 7E). *PIF4* expression in *elf3-13* and *elf3-14* are closer to WT, but the phase of expression is slightly advanced, consistent with their short period, and expression rises markedly during the late night, especially in *elf3-14* (Figure 7E). These findings are consistent with the phenotypes of these two hypomorphic alleles, with *elf3-14* hypocotyls slightly longer than *elf3-13*, correlating with the greater derepression of PIF4 in *elf3-14* (Figure 7E).

Taken together, these results indicate that the P666 and A37 residues are crucial for the normal developmental and circadian functions of ELF3 protein. Both mutations strongly diminish ELF3 activity and regulation of circadian clock output pathways and hypocotyl elongation, but significant functionality is retained, as evidenced by circadian oscillations for 4 days or more in *elf3-13* and *elf3-14* (Figure 4). *elf3-13* exhibits a shorter circadian period and greater degree of derepression of *PRR7* and *GI* than *elf3-14*, suggesting it is the stronger of the two mutant alleles with respect to clock function.

**FIGURE 8:**
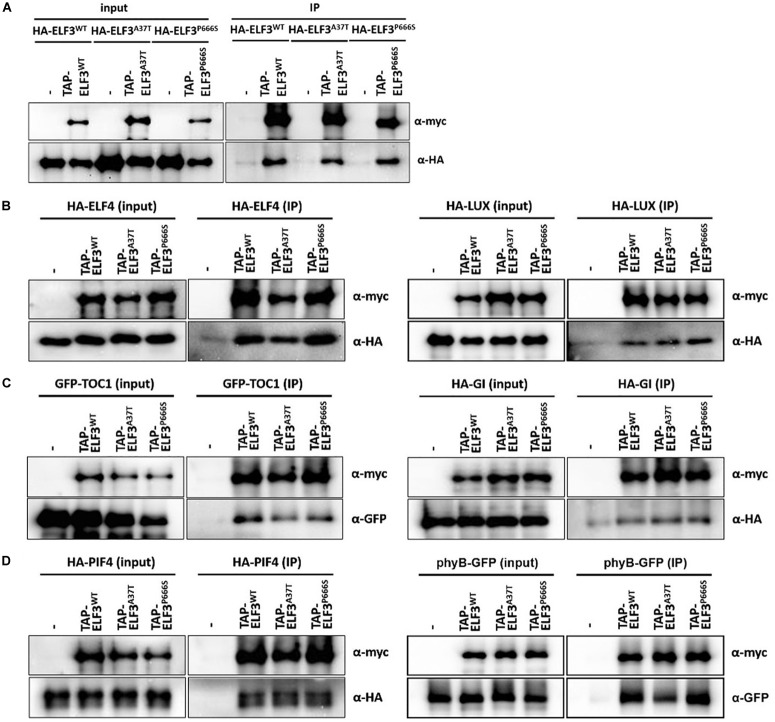
Interaction of *elf3* mutant alleles with select known interaction partners. TAP-ELF3^WT^, TAP-ELF3^A37T^ (elf3-14), and TAP-ELF3^P666S^ (elf3-13) proteins were co-expressed with either ELF3, ELF4, LUX, PIF4, phyB, GI, or TOC1, tagged with HA or GFP, in *N. benthamiana*. Infiltrated leaves were processed, total proteins were immunoprecipitated (human IgG) and precipitates were probed for the appropriate partner. **(A)** Homodimerization of ELF3 alleles. **(B)** Interactions with EC partners. **(C)** Interactions with TOC1 and GI. **(D)** Interactions with PIF4 and phyB. Representative immunoblots from two to three biological trials. Quantitation of these interactions are shown in Supplementary Figure S3.

To better understand how *elf3-13* (P666S) and *elf3-14* (A37T) compromise clock and hypocotyl function, we tested their interactions with known protein partners, including ELF3, ELF4, LUX, GI, TOC1, phyB, and PIF4 (Liu et al., 2001; Yu et al., 2008; Nusinow et al., 2011; Herrero et al., 2012; Nieto et al., 2015; Huang et al., 2016). We performed co-infiltration into *N. benthamiana* of *Agrobacterium* harboring appropriate pair-wise combinations of TAP-tagged ELF3 (WT, *elf3-13*, *elf3-14*) with HA-tagged or GFP-tagged ELF3, LUX, TOC1, GI, phyB, and PIF4 (Figure 8 and Supplementary Figure S3). Compared with WT ELF3, self-interaction for the two alleles appeared unaffected (Figure 8A and Supplementary Figure S3), though there is a weak statistically significant reduced interaction between WT ELF3 and elf3-14 (Supplementary Figure S3). The two other components of the EC, ELF4, and LUX also showed no detectable changes in their interaction with elf-13 or elf-14 protein (Figure 8B and Supplementary Figure S3).

TIMING OF CAB EXPRESION 1 has been recently identified as an ELF3 interactor, though the significance is unclear (Huang et al., 2016). The GI-ELF3 interaction has been implicated in the COP1-dependent turnover of GI protein (Yu et al., 2008). Neither *elf-13* nor *elf3-14* altered interactions with TOC1 or GI (Figure 8C and Supplementary Figure S3).

Phytochrome Interacting Factor4 (PIF4) transcript levels are regulated by ELF3 through its participation in the EC. ELF3 can also regulate PIF4 activity through direct binding to the PIF4 bHLH domain, suppressing *PIF4* transcriptional activity (Nieto et al., 2015). PIF4 interactions with ELF3 were not detectably altered by the *elf-13* and *elf3-14* mutations (Figure 8D and Supplementary Figure S3). phyB interacts with ELF3 as a likely point of intersection between light signaling and the circadian system (Huang and Nusinow, 2016; Huang et al., 2016). The *elf3-14* mutation reduced the interaction with phyB by half, while the *elf3-13* mutation had no effect (Figure 8D and Supplementary Figure S3). The A37T mutation resides within the known ELF3-phyB interaction domain [aa 1–201; (Liu et al., 2001)], suggesting we have identified a key ELF3 residue important in the phyB-ELF3 association.

## Discussion

Here we have characterized three new alleles of *ELF3* recovered from a forward genetic screen for PRR7 protein turnover factors. The approach relied on changes in the bioluminescence oscillation pattern of PRR7-LUC protein. Higher levels of PRR7-LUC during normal trough times could indicate a more stable protein, suggesting a loss-of-function in PRR7 proteolytic factors. This was observed in *ztl* mutants where strongly flattened rhythms of SCF^ZTL^ complex targets, TOC1 and PRR5, were seen under LD (Mas et al., 2003; Kiba et al., 2007; Fujiwara et al., 2008). Subsequent studies found that other members of the ZTL family (LKP2 and FKF1) also contribute to TOC1 and PRR5 turnover, but their contribution is less substantial than ZTL (Fujiwara et al., 2008; Baudry et al., 2010).

Reliance on changes in the PRR7-LUC waveform as the primary criterion meant that false positives that altered clock activity in ways unrelated to PRR7 proteolysis could be recovered, since the transgene was driven by the *PRR7* promoter. We observed that the flattened circadian oscillations of many mutants (Figure 2) were reminiscent of *elf3*, *elf4*, and *lux* null mutants (Hicks et al., 1996; Doyle et al., 2002; Hazen et al., 2005; Onai and Ishiura, 2005; McWatters et al., 2007). Sequencing these loci for all 20 surviving candidates revealed three novel *ELF3* alleles and one novel *ELF4* allele (translation termination). No mutants at the *LUX* locus were identified.

The *elf3-13* and *elf3-14* mutants are only the second and third alleles reported that retain significant but compromised function for multiple ELF3 controlled processes (Kolmos et al., 2011). In these mutants, the three primary developmental phenotypes affected by ELF3, flowering time, clock function and hypocotyl expansion are clearly intermediate between wild-type function and loss-of-function. Interestingly, all three processes are similarly compromised despite the location of the two mutations at opposite ends of the protein. Previous work suggested the N-terminal region, location of the A37T substitution of *elf3-14*, as important in mediating interactions with GI and phyB (Liu et al., 2001; Yu et al., 2008). Both GI and phyB play roles in flowering time, circadian period control and hypocotyl expansion (Koornneef et al., 1980; Goto et al., 1991; Somers et al., 1998; Park et al., 1999; Huq et al., 2000; Sawa et al., 2007). While the elf3-14-GI interaction is similar to WT ELF3-GI, the phyB-elf3-14 interaction is strongly reduced (Figure 8C). The phyB-ELF3 interaction domain maps to the N-terminus of ELF3, consistent with the location of the elf3-14 A37T mutation (Liu et al., 2001). The *elf3* and *phyB* mutants are similar in displaying long hypocotyls in red light and early flowering in long days (Liu et al., 2001). We have not measured the protein levels of phyB or ELF3 in the *elf3-14* background, but assuming they are normal, their reduced interaction suggests that a phyB-ELF3 association is key to normal hypocotyl development and flowering time. Our interaction assay was performed using tissue harvested under white light, and further extraction and immunoprecipitation also in the light. Previous *in vitro* work indicated that the phyB-ELF3 interaction is similar for both the Pr and Pfr forms of phytochrome (Liu et al., 2001). This suggests that the primary interaction is light-independent, but light conditions could still affect the recruitment of additional factors which are dependent on the phyB form. One possibility includes members of a MUT9-LIKE KINASE clade (MLKs) that associate with ELF3 in a phyB-dependent way (Huang et al., 2016).

Residue P666 resides near the C-terminus, in the PIF4 interaction domain (Nieto et al., 2015), although the P666S mutation (*elf3-13*) shows no detectable difference in the PIF4-elf3-13 interaction (Figure 8). However, this interaction is not relevant to the compromised repression of PIF4 and other genes (Figure 7), since all known transcriptional repressive activities of ELF3 are associated with its participation in the EC (Huang and Nusinow, 2016). ELF3 is thought to function as a scaffold, linking ELF4 with LUX, the DNA-binding partner of the tripartite complex (Huang and Nusinow, 2016; Huang et al., 2016). Our findings indicate that neither P666S nor A37T alters ELF3 binding to either partner, suggesting no effect on EC formation. However, modifications of the structure of the complex, which might alter recruitment of EC-interaction factors, or chromatin residence, may result from either or both mutations. The MLK kinases that associate with the EC (Huang et al., 2016) suggests a possible role for phosphorylation in the control of EC activity, co-factor interactions or chromatin binding. Both mutations add a potential phosphorylation site (S/T) which could result in an aberrant ELF3 phosphorylation state. ELF3 chromatin IPs in these *elf* mutant backgrounds would test one of these possibilities.

Also untested is the effect of *elf3-13* and *elf3-14* mutations on ELF3 nucleocytoplasmic distribution. ELF3 level in the nucleus is a key determinant in the effectiveness of ELF3 function in the clock (Herrero et al., 2012; Anwer et al., 2014). Nuclear localization of ELF3 is facilitated by ELF4 (Herrero et al., 2012), The ELF3-ELF4 interaction domain has been mapped to the middle of ELF3, exclusive of the A37 and P666 residues (Herrero et al., 2012), so it is not surprising that the neither mutation affects this interaction. However, nucleocytoplasmic partitioning is a multi-factor process and there are multiple mechanisms and proteins involved. Interactions with key nuclear import or nuclear exclusion partners might be affected by these *elf3* mutations (Meier and Somers, 2011; Floch et al., 2014). Examination of elf3-P666S and elf3-A37T nucleocytoplasmic distribution would test this hypothesis.

While our screen identified these new *elf3* alleles, three uncharacterized lines with similar PRR7-LUC profiles (e.g., Figure 2, pool#33) and no mutations in EC components or *GI* remain. Our forward genetics approach has demonstrated utility in uncovering novel reagents useful in probing the mechanics of the clock.

## Author Contributions

YJK and DS designed the research, analyzed the data, and wrote the manuscript. YJK performed the research.

## Conflict of Interest Statement

The authors declare that the research was conducted in the absence of any commercial or financial relationships that could be construed as a potential conflict of interest.
