# Corrigendum: Luciferase-Based Screen for Post-Translational Control Factors in the Regulation of the Pseudo-Response Regulator PRR7

**DOI:** 10.3389/fpls.2019.01057

**Published:** 2019-08-22

**Authors:** Yeon Jeong Kim, David E. Somers

**Affiliations:** Department of Molecular Genetics, The Ohio State University, Columbus, OH, United States

**Keywords:** circadian clock, pseudo-response regulator, PRR7, ELF3, ELF4, EMS mutagenesis, post-translational regulation

There is an error in the *Funding* statement. The correct number for the “Next-Generation BioGreen21 Program” is “PJ01327305.”A correction has therefore been made to the *Funding* statement:

“This work was supported by National Institutes of Health grant R01GM093285 and by a grant from the Next-Generation BioGreen21 Program (Systems and Synthetic Agrobiotech Center, Project PJ01327305), Rural Development Administration, Republic of Korea (DS).”

The authors apologize for this error and state that this does not change the scientific conclusions of the article in any way. The original article has been updated.

