# Supplementary material for: Luciferase-Based Screen for Post-translational Control Factors in the Regulation of the Pseudo-Response Regulator PRR7
Source: Front Plant Sci. 2019 May 22;10:667. doi: 10.3389/fpls.2019.00667 (PMC6540683; doi:10.3389/fpls.2019.00667)
Supplement: Supplementary file 1 [file Image_1.pdf]

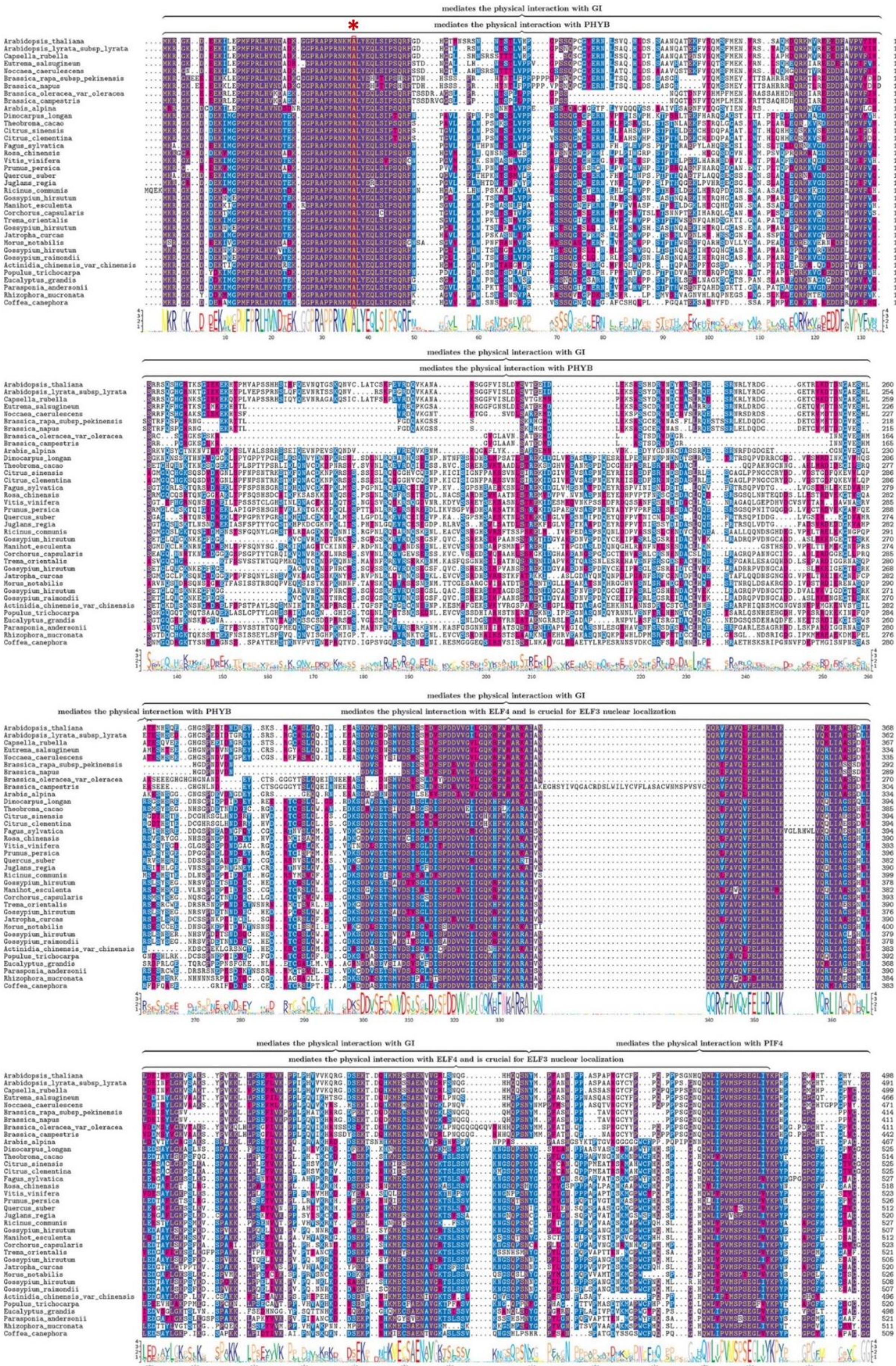
mediates the physical interaction with GI
mediates the physical interaction with PHYB
mediates the physical interaction with ELF4 and is crucial for ELF3 nuclear localization
mediates the physical interaction with PIF4
Arabidopsis_thaliana
Arabidopsis_lyrata_subsp_lyrata
Capsella_rubella
Eutrema_salsugineum
Noccaea_caerulescens
Brassica_rapa_subsp_pekinensis
Brassica_napus
Brassica_oleracea_var_oleracea
Brassica_campestris
Arabis_alpina
Dimocarpus_longan
Theobroma_cacao
Citrus_sinensis
Citrus_clementina
Fagus_sylvatica
Rosa_chinensis
Vitis_vinifera
Prunus_persica
Quercus_suber
Juglans_regia
Ricinus_communis
Gossypium_hirsutum
Manihot_esculenta
Corchorus_capsularis
Trema_orientalis
Jatropha_curcas
Morus_notabilis
Gossypium_raimondii
Actinidia_chinensis_var_chinensis
Populus_trichocarpa
Eucalyptus_grandis
Parasponia_andersonii
Rhizophora_mucronata
Coffea_canephora

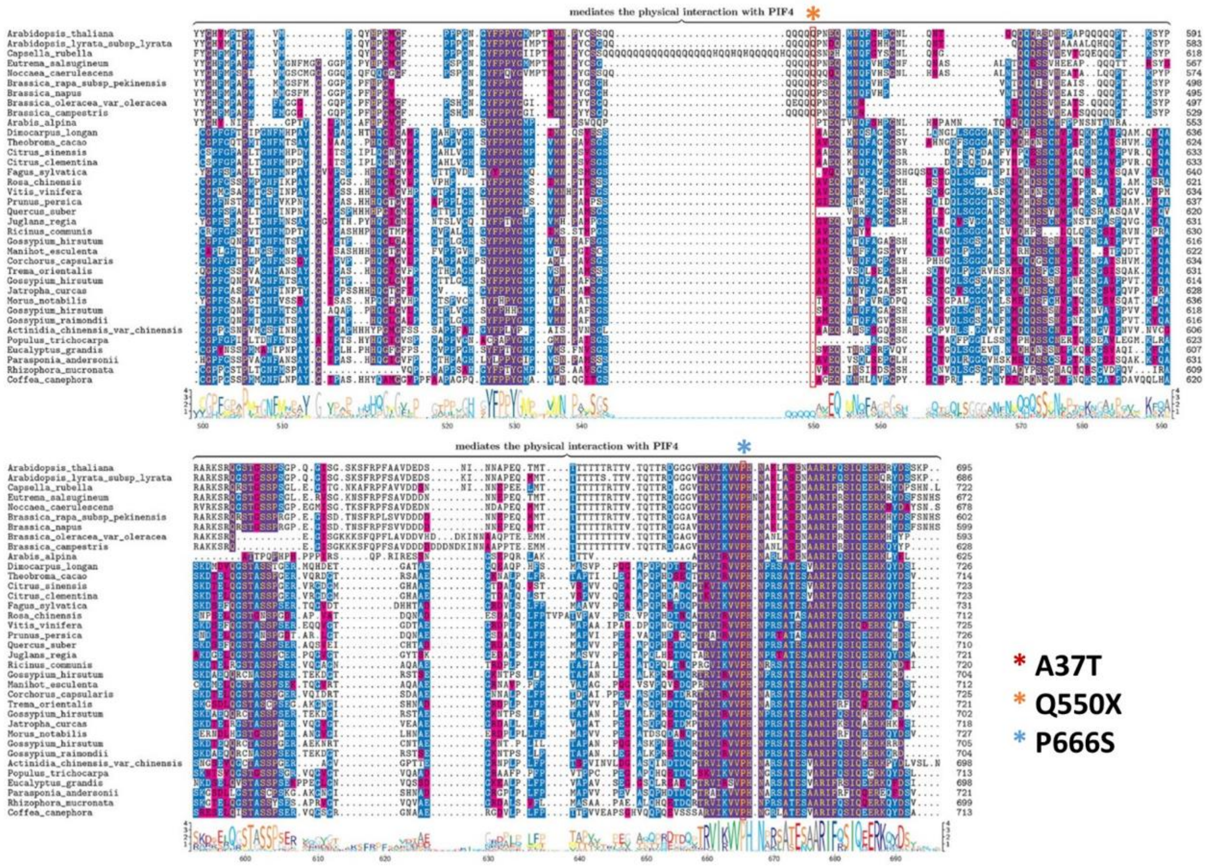

Supplementary Figure S1. Alignment of plant ELF3 homologs. Amino acid sequence of Arabidopsis ELF3 (AT2G25930.1) was used as query sequence in BLAST against the UniProtKB database (https://www.uniprot.org/). Among top 50 sequences (E-value cutoff 10^{-124}), one gene for each species, which were best matched with AtELF3, was selected and total 36 ELF3 orthologs were represented. The alignment image was rendered using TeXshade (Beitz, 2000). Note that the amino acid residues corresponding to the substitution mutations identified in this study (asterisks and red box) are highly conserved across the homologs. The known interaction regions of ELF3 are shown above the aligned sequences, if identified. Sources for delimiting the regions of interaction with known ELF3 partners are as follows: ELF3 homodimerization, PHYB (Liu et al., 2001); ELF4, LUX (Nusinow et al., 2011); PIF4 (Nieto et al., 2015); GI, COP1 (Yu et al., 2008).
