# Supplementary figures and images for: Luciferase-Based Screen for Post-translational Control Factors in the Regulation of the Pseudo-Response Regulator PRR7
Source: Front Plant Sci. 2019 May 22;10:667. doi: 10.3389/fpls.2019.00667 (PMC6540683; doi:10.3389/fpls.2019.00667)

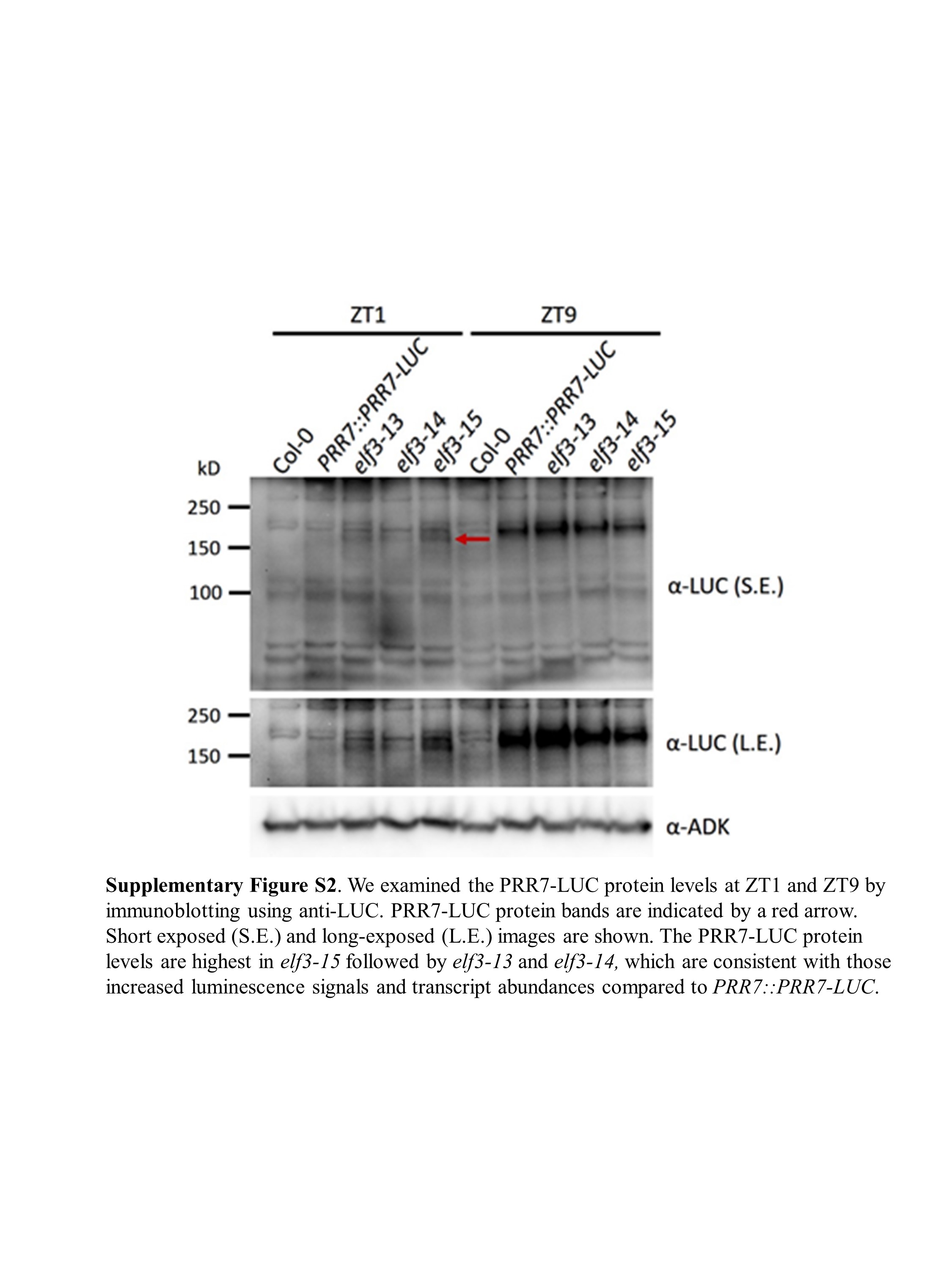

Supplement: Supplementary file 2 [file Image_2.jpeg]

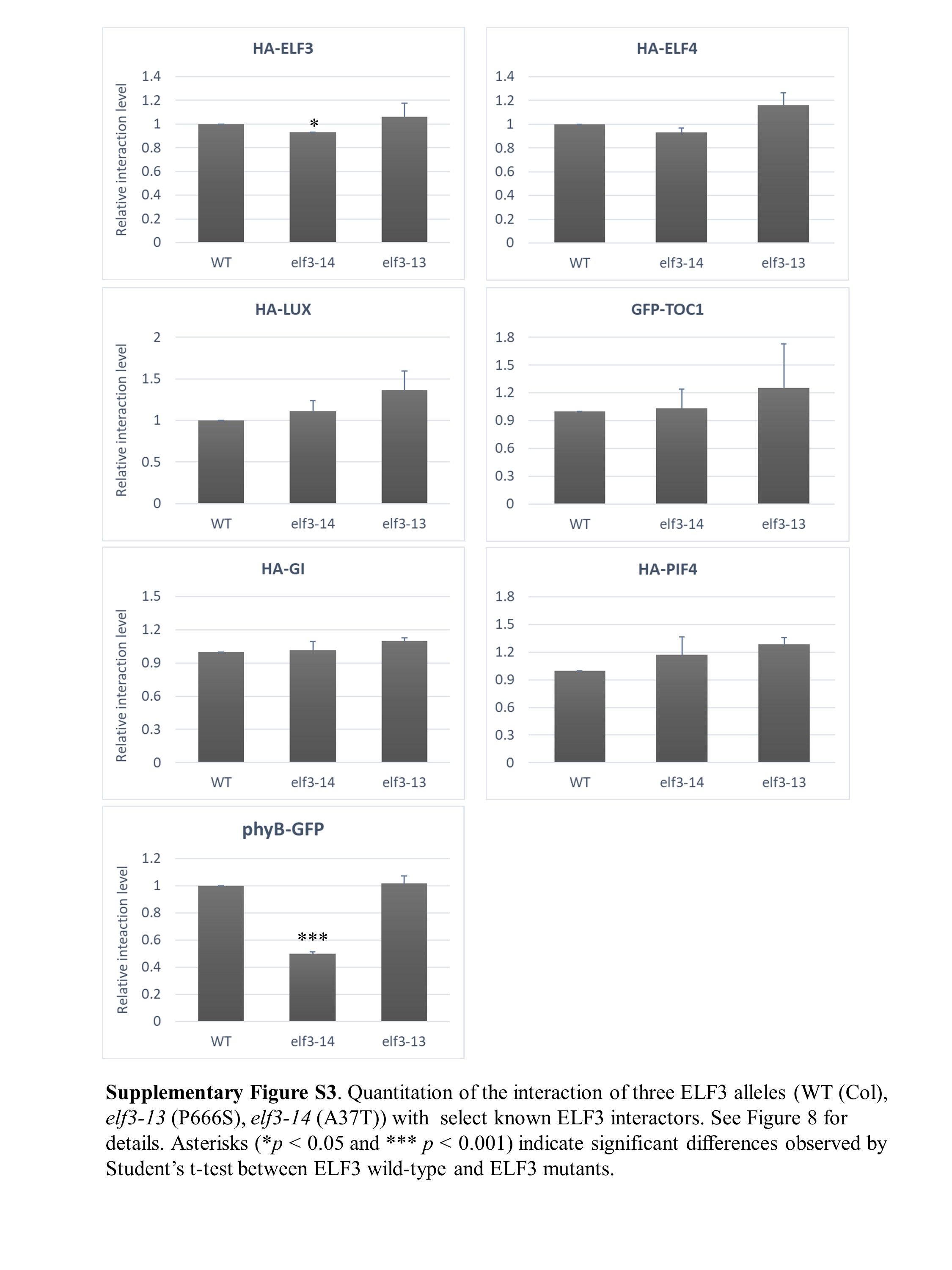

Supplement: Supplementary file 3 [file Image_3.jpeg]
