# Supplementary material for: Luciferase-Based Screen for Post-translational Control Factors in the Regulation of the Pseudo-Response Regulator PRR7
Source: Front Plant Sci. 2019 May 22;10:667. doi: 10.3389/fpls.2019.00667 (PMC6540683; doi:10.3389/fpls.2019.00667)
Supplement: Supplementary file 4 [file Data_Sheet_1.pdf]

Supplementary Table S1. Primers used for qRT-PCR in this study

Gene	Forward (5’ -> 3’)	Reverse (5’ -> 3’)
CCA1	TCTGGTTATTAAGACTCGGAAGCCAT	CTTCTGCCATGCTCTACCATAAAGCC
PRR9	CCTTGAAGATACTGATGAAACTTGTG	TGTAATTGATAGCCTGAGCACCACTT
PRR7	ACGACTGAGAACAACGCTTTCACAAA	ACATGGACAAGGTTATAGGAGGCATG
GI	AATTCAGCACGCGCCTATTG	GTTGCTTCTGCTGCAGGAACTT
PIF4	ACCTCAGAGACGGTTAAGCC	TGGAGGAGGCATGACTTGAG
U-box	TGCGCTGCCAGATAATACACTATT	TGCTGCCCAACATCAGGTT

Primers used for plasmid construction in this study

Gene	Forward (5’ -> 3’)	Reverse (5’ -> 3’)
PRR7pro	ATCGATATCCCTTTTGCAGTCACGATGAT	GTAGATATCCACACCAACTCTGCTTCGCT
PRR7	GCTACCATGGATGAATGCTAATGAGGAG GG	TAGCCCATGGCGCTATCCTCAATGTTTTTTA
TAP-ELF3	CACCGCATGAAGAGAGGGAAAGATGAG G	TTAAGGCTTAGAGGAGTCA
HA-ELF3	CACCATGAAGAGAGGGAAAGAT	TTAAGGCTTAGAGGAGTCA
ELF3^{A37T}	AGAAACAAGATGACTCTTTATGAGCAG	CTGCTCATAAAGAGTCATCTTGTTTCT
ELF3^{P666S}	ATAAAGGTGGTATCTCACAACGCAAAG	CTTTGCGTTGTGAGATACCACCTTTAT
ELF4	CACCATGAAGAGGAACGGCGAG	TTAAGCTCTAGTTCCGGC
LUX	CACCATGGGAGAGGAAGTACAAA	ATTCTCATTTGCGCTTCC
GI	CACCATGGCTAGTTCATCTTCA	TTATTGGGACAAGGATATAG
PIF4	CACCATGGAACACCAAGGTTGGAG	CTAGTGGTCCAAACGAGAACCG
